# Vascular catheter colonization: surveillance based on culture of needleless connectors

**DOI:** 10.1186/s13054-016-1334-1

**Published:** 2016-05-28

**Authors:** María Jesús Pérez-Granda, María Guembe, Raquel Cruces, Emilio Bouza

**Affiliations:** Department of Clinical Microbiology and Infectious Diseases, Hospital General Universitario Gregorio Marañón, Madrid, Spain; Cardiac Surgery Postoperative Care Unit, Hospital General Universitario Gregorio Marañón, Madrid, Spain; Instituto de Investigación Sanitaria Gregorio Marañón (IiSGM), Madrid, Spain; Medicine Department, School of Medicine, Universidad Complutense de Madrid, Madrid, Spain; CIBER de Enfermedades Respiratorias-CIBERES (CB06/06/0058), Madrid, Spain; Hospital General Universitario “Gregorio Marañón”, C/Dr. Esquerdo, 46, 28007 Madrid, Spain

**Keywords:** Needleless connectors, Flushing, Superficial culture, Hub culture, Catheter colonization, Catheter-related bloodstream infection

## Abstract

**Background:**

Superficial culture has a high negative predictive value in the assessment of catheter tip colonization (CC) and catheter-related bloodstream infection (C-RBSI). However, the process of hub culture requires the hubs to be swabbed, and this carries a risk of dislodging the biofilm. At present, most catheter hubs are closed by needleless connectors (NCs) that are periodically replaced. Our objective was to compare the yield of SC (skin + hub culture) with that of skin + NC culture in the assessment of CC and C-RBSI.

**Methods:**

During 5 months, we included the patients on the Major Heart Surgery ICU when a central venous catheter (CVC) remained in place ≥7 days after insertion. SCs were taken simultaneously when the NC was withdrawn and processed by the semi-quantitative method, even when the catheter was not removed. All catheter tips were cultured. All NCs belonging to a single catheter lumen were individually flushed with 100 μl of brain-heart infusion (BHI) broth. We considered the lumen to be colonized when ≥1 NC culture from the lumen flush was positive. We collected a total of 60 catheters.

**Results:**

The overall CC rate was 15.0 %, and we confirmed two episodes of C-RBSI. The validity values after the comparison of SCs with skin + NC culture for prediction of CC were the following: sensitivity 66.7 % vs. 77.8 %, and negative predictive value 93.6 % vs. 93.1 %. The sensitivity and negative predictive value for prediction of C-RBSI was 100 % for both SC and skin + NC culture.

**Conclusion:**

The combination of skin and flushed NC culture can be an alternative to conventional SC for ruling out CC and C-RBSI.

## Background

The conventional conservative diagnostic method based on superficial culture requires a surface culture of the skin surrounding the catheter insertion site and a surface culture of the inside of each catheter hub [1]. This procedure has been used in several populations to select patients at risk of catheter-related bloodstream infection (C-RBSI) and to rule out the catheter as the source of the bloodstream infection [2–6]. However, the hub culture process requires needleless connectors (NCs) to be removed so that the swab cab be rubbed along the inside of the lumen. The consequent manipulation of the hub can dislodge the biofilm and potentially trigger bloodstream infection [7–9].

A recent study by our group showed that culture (by instillation of brain-heart infusion broth) of the NCs used to close the catheter hubs combined with skin culture, can be an alternative and safer procedure for prediction of catheter colonization and ruling out C-RBSI [10]. However, data on superficial hub culture were not available and, therefore, the yield of conventional superficial culture (skin + hubs) could not be compared with that of skin + NC cultures for prediction of catheter colonization and C-RBSI. The aim of the present study was to compare the validity values of conventional superficial culture (skin + hubs) with those of skin + NC cultures for catheter tip colonization and C-RBSI.

## Methods

### Setting

The Major Heart Surgery ICU (MHS-ICU) in our hospital is a 14-bed post-surgical unit for all adult patients who have undergone a major cardiac surgical procedure. Patients admitted to the MHS-ICU during the study period (2 July 2015 to 23 October 2015) were included in the study when a central catheter remained in place ≥7 days after insertion.

### Laboratory procedure

**Fig. 1:**
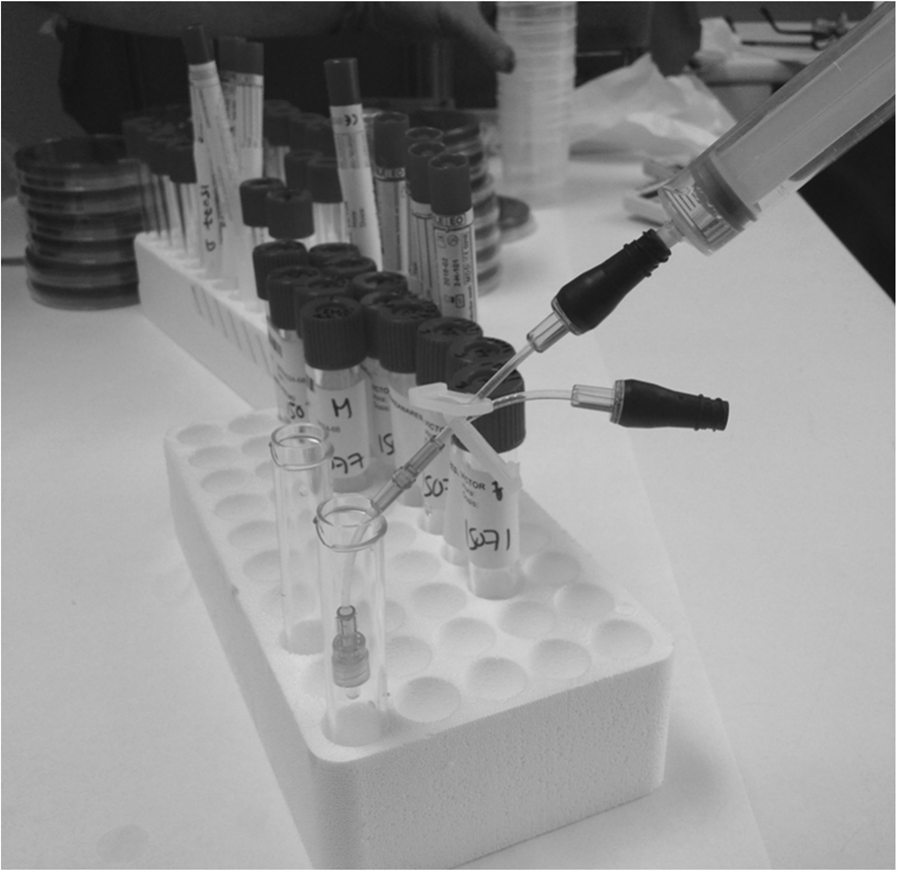
Laboratory procedure for needleless connector (NC) flushing

Following the manufacturer’s instructions, NCs (CLAVE™ systems, ICU Medical, Inc., San Clemente, CA, USA) were changed every 7 days and cultured. Simultaneously, superficial culture (from the skin surrounding the catheter insertion site and from the inside of the hubs) was also taken when the NC was withdrawn, even when the catheter was not removed. When the catheter was removed, the last set of NCs and hub cultures was also obtained. Superficial cultures were processed following standard semi-quantitative microbiological techniques [1]. All groups of NCs belonging to a single catheter lumen were individually flushed with 100 μl of brain-heart infusion broth (Fig. 1). We considered the lumen colonized when ≥1 culture was positive. The number of cultured NCs varied depending on the number of lumens in each catheter (1–5 lumens).

Catheter tips were withdrawn when clinically indicated, and upon withdrawal they were cultured using the roll-plate (Maki) technique and sonicated onto a blood agar plate [11]. The microorganisms recovered were identified using standard microbiological methods and MALDI-TOF [12].

We also followed a pre-established protocol to record patient characteristics, underlying diseases, comorbidity factors, severity of illness scores (e.g., acute physiology and chronic health evaluation (APACHE) II), the maximum severity reached until catheter withdrawal, and microbiological data from blood cultures.

### Definitions

#### Catheter tip colonization

Isolation of either ≥15 colony forming units (cfu)/plate using Maki’s semi-quantitative technique or ≥100 cfu/catheter by sonication.

#### Skin colonization

Isolation of ≥15 cfu/plate by semi-quantitative culture.

#### Hub colonization

Isolation of ≥15 cfu/plate by semi-quantitative culture.

#### Closed needleless connector colonization

Isolation of ≥1 cfu/plate in at least one connector in the qualitative culture.

#### Overall lumen colonization

When ≥1 NC became positive at any point during surveillance.

#### C-RBSI

We considered a C-RBSI episode to be confirmed when the same microorganism was isolated both in peripheral blood cultures and in the catheter tip. The gold standard for confirmation of catheter colonization was positivity of the catheter tip culture either using Maki’s semi-quantitative technique (≥15 cfu/plate) or by sonication (≥100 cfu/segment). To calculate the validity values of superficial culture (skin + hub) and skin + NC culture for prediction of catheter colonization, we used a positive catheter tip with ≥15 cfu/plate of any microorganism as the gold standard.

### Statistical analysis

Values are expressed as the mean (SD) or median (IQR) for continuous variables and as percentages, with the 95 % confidence interval (95 % CI), when applicable, for categorical variables. Categorical variables were evaluated using the chi-square test or the two-tailed Fisher exact test. Statistical significance was set at *p* < 0.05 (two-tailed). We calculated the validity values of the superficial culture (skin + hubs) and skin + NC culture by comparing them with the gold standard of colonization and also with the results obtained by culture of the superficial samples. The sensitivity, specificity, and positive and negative predictive values, with 95 % CI, were calculated using EPIDAT 3.1. Accuracy was defined as the sum of true positive and true negative results. The power analysis was calculated for the negative predictive value and sensitivity. Statistical analysis was performed using IBM SPSS Statistics for Windows, Version 21.0 (IBM Corp, Armonk, NY, USA).

### Ethics

The study was approved by the local ethics committee of Hospital General Universitario Gregorio Marañón and the ethics committee waived the need for informed consent.

## Results

We collected 60 catheters from 34 patients who had a catheter inserted for ≥7 days. The mean (SD) age was 63.7 (11.9) years. The main underlying conditions were congestive heart failure (67.6 %) and diabetes mellitus (35.3 %). The mean (SD) comorbidity index, APACHE II at inclusion, and EuroScore were 2.6 (1.7), 8.3 (3.3), and 6.7 (2.5), respectively. The main reason for catheter withdrawal was end of use (63.3 %), followed by suspicion of infection (25.0 %), and other reasons (11.7 %). We confirmed 2 episodes of C-RBSI (2.2 episodes/1,000 catheter days). Other patient and catheter data are detailed in Table 1. The crude mortality rate of the study population was 26.7 %.

**Table 1:** Main patient and catheter characteristics

Characteristic	N (%)
Patients (N=34)	
Mean (SD) age, years	63.7 (11.9)
Sex, male/female	21/13
Underlying conditions (%)	
Myocardial infarction	3 (8.8)
Congestive heart failure	23 (67.6)
ACVA	6 (17.6)
Chronic obstructive pulmonary disease	7 (11.7)
Diabetes mellitus	12 (35.3)
Peptic ulcer disease	6 (17.6)
Peripheral vascular disease	3 (8.8)
Renal dysfunction	7 (20.6)
EuroScore^a^, mean (SD)	6.7 (2.5)
Charlson comorbidity index, mean (SD)	2.6 (1.7)
Non-fatal underlying disease, McCabe (%)	25 (73.5)
APACHE II at inclusion, mean (SD)	8.3 (3.3)
Length of ICU stay (days), median (IQR)	10.5. (7.7-38.0)
Crude mortality (%)	8 (26.7)
Catheters (N=60)	
Type	
Non-tunneled central venous catheter	47 (78.3)
Guidewire	13 (21.7)
Location	
Jugular	56 (93.3)
Subclavian	4 (6.7)
Total parenteral nutrition/propofol	17 (28.3)
Reasons for catheter withdrawal	
End of use	38 (63.3)
Suspicion of infection	15 (25.0)
Others	7 (11.7)
Catheter-days, median (IQR)	11.0 (8.0-20.0)
Total catheter-days	906
Catheter colonization (%)	9 (15.0)
Catheter colonization/1,000 catheter-days	9.9/1,000
C-RBSI episodes (%)	2 (3.3)
C-RBSI/1,000 catheter-days	2.2/1,000

SD, standard deviation; IQR, interquartile range; ICU, intensive care unit; ACVA, acute cerebrovascular accident; C-RBSI, catheter-related bloodstream infection

^a^ EuroScore, European System for Cardiac Operative Risk Evaluation

The overall catheter tip colonization rate was 15.0 % (9/60). Out of the 60 catheters, 29 (48.3 %) skin and/or NC and/or hub cultures were never positive. In the remaining 31 (51.7 %), skin and/or NCs and/or hubs were positive at least once.

Table 2 shows the validity values for skin + NC cultures and superficial culture for prediction of catheter colonization and C-RBSI. Skin + NC cultures had 77.8 % sensitivity and a 93.1 % negative predictive value for catheter colonization compared with 66.7 % and 93.6 % for superficial culture (*p* = 0.02). The accuracy for skin + NC cultures and superficial culture was 7 true positive/27 true negative, and 6 true positive/44 true negative, respectively. NC cultures had the same negative predictive value for C-RBSI (100 %). Table 3 shows the microorganisms isolated from the colonized central venous catheters.

**Table 2:** Validity values of skin and NC culture and superficial culture (skin + hubs) for prediction of catheter colonization and C-RBSI

CULTURE	S% 95% CI	SP% 95% CI	PPV% 95% CI	NPV% 95% CI	Validity index 95% CI	Prevalence 95% CI	LR+ 95% CI	LR- 95% CI
Catheter colonization								
Skin+NCs	77.8 (45.0-100)	52.9 (38.3-67.6)	22.6 (6.2-38.9)-	93.1 (82.2-100)	56.7 (43.3-70.0)	15.0 (5.1-24.9)	1.65 (1.05-2.60)	0.42 (0.12-1.46)
Skin+hubs	66.7 (30.3-100)	86.3 (75.8-92.0)	46.1 (15.2-77.1)	93.6 (85.6-100)	83.3 (73.0-93.6)	15.0 (5.1-24.9)	4.86 (2.12-11.13)	0.39 (0.15-0.98)
C-RBSI								
Skin+NCs	100 (75.0-100)	50.0 (36.3-63.7)	6.4 (0.0-16.7)	100 (98.3-100)	51.7 (38.2-65.1)	3.3 (0.0-8.7)	2.00 (1.55-2.59)	NA
Skin+hubs	100 (75.00-100)	81.0 (70.0-91.9)	15.4 (0.0-38.8)	100 (98.9-100)	76.8 (67.0-86.6)	3.3 (0.0-8.7)	5.27 (3.10-8.98)	NA

S, sensitivity; SP, specificity; PPV, positive predictive value; NPV, negative predictive value; LR+, positive likelihood ratio; LR-, negative likelihood ratio; CI, confidence interval; NA, not applicable; C-RBSI, catheter-related bloodstream infection

**Table 3:** Microorganisms isolated in colonized catheters

Catheter tip	Skin + NCs	Skin + hubs
*Staphylococcus epidermidis*	*Staphylococcus epidermidis*	*Staphylococcus epidermidis*
*Staphylococcus epidermidis*	*Staphylococcus epidermidis*	*Staphylococcus epidermidis*
*Staphylococcus epidermidis*	*Staphylococcus epidermidis*	*Staphylococcus epidermidis*
CoNS	*-*	*-*
*Staphylococcus epidermidis*	*Staphylococcus epidermidis* *Moraxella osloensis* *Klebsiella pneumoniae*	*Staphylococcus epidermidis* *Klebsiella pneumoniae*
*Candida albicans*	*Staphylococcus hominis*	*-*
*Staphylococcus epidermidis*	*Staphylococcus epidermidis* *Staphylococcus aureus* *Staphylococcus saprophyticus*	*Staphylococcus epidermidis*
*Staphylococcus epidermidis*	*Staphylococcus epidermidis*	*Staphylococcus epidermidis*
*Staphylococcus hominis*	*-*	*-*

NCs, needleless connectors; CoNS, coagulase-negative staphylococci

## Discussion

The new procedure we describe for ruling out catheter colonization and C-RBSI based on the combination of skin and NC cultures showed, at least, no inferiority to the conventional superficial culture. Guidelines recommend using superficial culture (skin and hub) as a useful conservative procedure for the diagnosis of catheter tip colonization and C-RBSI [1]. The recommendation is based on the pathogenesis of catheter colonization, which occurs by progression of microorganisms to the tip of the catheter along either the inner surface (≥7 days of indwelling time) or the outer surface (<7 days of indwelling time) of the catheter. Superficial culture has been used in several populations (e.g., MHS-ICU, oncology, and hemodialysis patients) and helps prevent unnecessary catheter withdrawal [2–6]. However, conventional superficial culture requires swabs to be rubbed along the inside of the hubs, thus increasing the risk of dislodging the biofilm and triggering bloodstream infection [7–9].

Our group recently tested an alternative superficial culture procedure for hubs [10]. We hypothesized that as NCs are substituted periodically to decrease catheter colonization, they can be used as an alternative to hub culture for diagnosis, thus avoiding excessive manipulation. The laboratory procedure was performed by flushing 100 μl of brain-heart infusion broth through the inside of the NCs before culture. The results showed that NCs combined with skin superficial culture had good sensitivity and negative predictive values for catheter colonization and C-RBSI. However, data were not compared with those for superficial hub culture, as this approach was not used.

In the present study, we resolved this limitation and found that the sensitivity for predicting catheter colonization using skin + NC culture compared with conventional superficial culture was 77.8 % vs. 66.7 % (*p* = 0.02). However, both combinations of procedures had better accuracy for the coagulase-negative Staphylococci than for the other etiological origins. Besides, we did not find statistically significant differences between the procedures in the prediction of C-RBSI. The most important finding was that skin + NC culture had a high negative predictive value for catheter colonization and C-RBSI (93.1 % and 100 %, respectively).

The main limitation of the study was the small sample size and the low rate of catheter tip colonization. However, we calculated the power analysis for the negative predictive value and sensitivity of the obtained results as follows: negative predictive value 4.0 % (93.1–93.6 %); sensitivity 28.0 % (77.8–66.7 %). The obtained power for sensitivity was enough to detect statistical significant differences (*p* = 0.02). We consider that our results are promising for the substitution of superficial hub culture by culture of NC instillations. Future clinical studies are needed to assess the impact of this procedure in the prevention of catheter tip colonization.

## Conclusions

We demonstrated that superficial skin culture combined with NC culture was not inferior to conventional superficial culture of skin and hubs in the selection of patients with no risk of catheter colonization or C-RBSI. Moreover, the new diagnostic approach is easier and less invasive to apply.
